# Clinicians' Behavior Toward Radiology Reports: A Cross-Sectional Study

**DOI:** 10.7759/cureus.11336

**Published:** 2020-11-05

**Authors:** Azza S Reda, Dalia Abdulmonem Hashem, Khalid Khashoggi, Felwa Abukhodair

**Affiliations:** 1 Diagnostic Radiology, King Abdulaziz University, Jeddah, SAU; 2 Information Technology, King Abdulaziz University, Jeddah, SAU

**Keywords:** reporting, physician, clinician, medical errors, communications, radiology report

## Abstract

Background

The radiology report is the way of communication between the radiologists and the clinicians of different specialties. Each part of the report is important and significant in the patient management plan. Therefore, knowledge of interpretation and behavior in understanding the final report is a variable crucial skill.

Methods

This is a cross-sectional survey study to explore the behavior and attitude of clinicians toward radiology reports in relation to their professional clinical demographic. A total of 107 physicians participated, including consultants, specialists, and residents among different specialties.

Results

Among the 107 responses, 58.9% were male and 41.1% were female. The majority of the physicians (78.5%) read the radiology report for every requested study for each patient, while 21.5% of participants didn’t read the radiology report for the studies they requested, instead, they only read it occasionally. Gender played a significant factor, as female practitioners were more likely to read the complete radiology report (P = 0.033). In addition, the age of the practitioner was also significant as clinicians in the age group 40-60 years old were more likely to check the requested radiology image prior to reading the report compared to age groups 20-39 and >60 years (P = 0.035). Lastly, specialists were significantly more likely to read the entire radiology report compared to consultants and residents (P = 0.006).

Conclusion

More emphasis and awareness should be provided to clinicians on the importance of reading the entire radiology report as some information can be missed if not being read completely.

## Introduction

The radiology report is considered the most important form of communication between the clinicians and the radiologist if not often to be the only method. It is also incorporated as a part of the patient’s medical records and plays an essential part in the way clinical care is approached. As the diagnostic process is becoming more complex, radiology reports take on an even more important role [1].

Many studies have evaluated the characteristics and the preferences of both radiologists and other clinicians with regard to radiology reports. Recently, a research study revealed that radiologists preferred a more detailed radiology report that was written in free text (i.e., unstructured), where it also incorporates mentioning of the examination techniques conducted by the radiologist [2]. Another study mentioned the preference of some imaging centers for radiology reports to be structured which would facilitate the access of information, teaching, clinical research, and other related aspects [3]. The aforementioned characteristics and many others form the basis of radiology reports and generate discrepancies regarding the opinions of the best model of a radiology report to be adopted. That being said, the opinion of the referring physician and forming the report in an accurate easy practical language is also of great importance to the process of refining radiology reports, given the fact that they are the final recipients of these reports [4].

In this setting, we conducted the current investigation to study clinicians’ behavior regarding the radiology report. We aim to determine how often do clinicians read the radiology report, which part do they most commonly read, how frequently do they check the radiology images before they read the text, and how frequently they make medical errors that could have been easily avoided by reading the report more carefully. This will help shed light on areas of improvement of radiology reporting, how to raise awareness among clinicians, and reduce potential errors that can be made from either incomplete report reading or misinterpretation.

## Materials and methods

This is a cross-sectional study, which was approved by the local ethics committee at King Abdul-Aziz University Hospital, Jeddah, Saudi Arabia. A multiple-choice questionnaire was elicited and constructed from previous similar studies [1,5,6]. The questionnaire was distributed electronically using the SurveyMonkey website and was distributed through emails to the physicians from 16 various specialties. The study sample included consultants, specialists, and resident physicians. The study was carried out from August 2019 to December 2019 and incomplete data were excluded from the final dataset.

The questionnaire included two parts. The first part consisted of four demographic questions which included gender, age, professional level, and specialty. The second part consisted of four multiple-choice questions about clinician behavior toward reading radiology reports. A pilot study was conducted, and the questionnaire showed reliability and validity value of Cronbach's alpha > 0.75. All 107 responders did not include any personal informations and were completely anonymous. The statistics were analyzed using Statistical Package for the Social Sciences (SPSS 24, IBM Corp., Armonk, NY). A chi-square test of independence was performed to examine the relationship between different independent categorical variables and a P-value of < 0.05 was considered significant.

## Results

Demographic characteristic

A total of 107 complete questionnaires were included in the final analysis step. Among the respondents, 63 (58.9%) were males and 44 (41.1%) were females. The majority of respondents (70.1%) were of the age group 20-39 years. Meanwhile, 44 (41.1%) participants were residents, 40 (37.4%) were consultants, and 23 (21.5%) were specialists. Included clinicians were of diverse specialties, the pediatric specialty was the most common (24.3%) followed by internal medicine (18.7%) and general surgery (14%). Baseline demographic characteristics of included participants are summarized in Table 1.

**Table 1:** Baseline demographic characteristics of included participants OB & Gyn: obstetrics and gynecology; ENT: ear, nose and throat; ICU: intensive care unit

Variable	Sub-group	Number	Percentage (%)
Gender			
	Male	63	58.9
	Female	44	41.1
Age group (years)			
	20-39	75	70.1
	40-60	30	28
	>60	2	1.9
Level			
	Consultant	40	37.4
	Specialist	23	21.5
	Resident	44	41.1
Specialty			
	General surgery	15	14
	OB & Gyn	8	7.5
	Orthopedic	5	4.7
	ENT	3	2.8
	ICU	1	0.9
	Pediatrics	26	24.3
	Internal medicine	20	18.7
	Anesthesia	3	2.8
	Emergency	10	9.3
	Neurosurgery	1	0.9
	Urology	2	1.9
	Cardiothoracic	2	1.9
	Plastic surgery	2	1.9
	Ophthalmology	1	0.9
	Family medicine	3	2.8
	Other	5	4.7

Behavior

A total of 84 clinicians (78.5%) read the radiology report for every requested examination, while 23 clinicians (21.5%) read the radiology report occasionally. On the other hand, the majority of respondents, 60 clinicians (56.1%), reported that they read the entire radiology report, while only 7 (6.5%) read the conclusion part. In the same context, the majority of respondents, 71 (66.4%), reported that they check the radiology images before reading the report itself, while 29 (27.1%) respondents reported checking the radiology images after reading the report. Although the majority of respondents, 68 (63.6%), never made a medical error that could have been prevented by reading the radiology report, 16 clinicians (15%) committed only one medical error that could have been prevented by reading the report. The same number of 16 clinicians (15%) made an error two to five times by not reading the entire report carefully. The behaviors of clinicians in reading the radiology images and reports are presented in Table 2.

**Table 2:** Clinicians’ behavior toward the radiology report

Variable	Sub-category	Number	Percentage
How often do you read the radiology report?			
	Every requested examination	84	78.5%
	Occasionally	23	21.5%
Which part of the radiology report you read?			
	Only conclusion	7	6.5%
	The entire report	60	56.1%
	The conclusion and scan through the text	40	37.4%
Do you check your requested radiology images?			
	No	6	5.6%
	Yes (before reading the report)	71	66.4%
	Yes (after reading the report)	29	27.1%
	Missing	1	0.9%
Have you ever made an avoidable medical error in patient care that could have been prevented by reading the radiology report more carefully?			
	No, never	68	63.6%
	Yes, once in my entire career	16	15%
	Yes, 2-5 times	16	15%
	Yes, >5 times	7	6.5%

The behaviors toward reading the radiology report were then stratified based on gender, age, level, and specialty. We found that females were significantly more likely to read the radiology report for every requested examination compared to males (88.60% vs 71.40%, P = 0.033). Meanwhile, both male and female respondents read the entire radiology report (55.60% vs 56.80%, P = 0.311). We noted no statistically significant differences in the frequency of checking the requested radiology images between both genders (P = 0.687) or the number of medical errors that could have been prevented by reading the radiology report more carefully (P = 0.803). Inferential data by gender is presented in Table 3.

**Table 3:** Stratification of clinicians’ behavior towards the radiology report by gender ^a^Chi-square test with P < 0.05 being statistically significant. ^b^Data for one participant was missing.

Variable	Sub-category	Total N (%)	Male N (%)	Female N (%)	P-value
How often do you read the radiology report?					
	Every requested examination	84 (78.5%)	45 (71.40%)	39 (88.60%)	0.033^a^
	Occasionally	23 (21.5%)	18 (28.60%)	5 (11.40%)	
Which part of the radiology report you read?					
	Only conclusion	7 (6.5%)	6 (9.50%)	1 (2.30%)	0.311
	The entire report	60 (56.1%)	35 (55.60%)	25 (56.80%)	
	The conclusion and scan through the text	40 (37.4%)	22 (34.90%)	18 (40.90%)	
Do you check your requested radiology images? ^b^					
	No	6 (5.6%)	3 (4.80%)	3 (7.00%)	0.687
	Yes (before reading the report)	71 (66.4%)	41 (65.10%)	30 (69.80%)	
	Yes (after reading the report)	29 (27.1%)	19 (30.20%)	10 (23.30%)	
Have you ever made an avoidable medical error in patient care that could have been prevented by reading the radiology report more carefully?					
	No, never	68 (63.6%)	38 (60.30%)	30 (68.20%)	0.803
	Yes, once in my entire career	16 (15%)	10 (15.90%)	6 (13.60%)	
	Yes, 2-5 times	16 (15%)	11 (17.50%)	5 (11.40%)	
	Yes, >5 times	7 (6.5%)	4 (6.30%)	3 (6.80%)	

Age had no significant factor on how often clinicians read the radiology report (P = 0.221) nor on the frequency of making avoidable medical errors by reading the whole report (P = 0.659). On the other hand, gender showed statistical significance toward the frequency of reading reports (P = 0.033) with females showing more tendencies to reading the report every time it was requested (Table 3). Clinicians whose age ranges from 40-60 years are significantly more likely to read the entire radiology report compared to age groups 20-39 and >60 years (70% vs 50.7% vs 50%, P = 0.031). In the same context, clinicians in both age groups 20-39 and >60 years compared with clinicians in the age group 40-60 years are significantly more likely to check the requested radiology image prior to reading the report (64% vs 0% vs 79.3%, P = 0.035) (Table 4).

**Table 4:** Stratification of clinicians’ behavior toward the radiology report by different age groups ^a^Chi-square test with P < 0.05 being statistically significant. ^b^Data for one participant was missing.

Variable	Sub-category	Age group (years) N: Numbers (%)			P-value
		20-39	40-60	>60	
How often do you read the radiology report?					
	Every requested examination	62 (82.70%)	21 (70.00%)	1 (50.00%)	0.221
	Occasionally	13 (17.30%)	9 (30.00%)	1 (50.00%)	
Which part of the radiology report you read?					
	Only conclusion	3 (4.00%)	3 (10.00%)	1 (50.00%)	0.031^a^
	The entire report	38 (50.70%)	21 (70.00%)	1 (50.00%)	
	The conclusion and scan through the text	34 (45.30%)	6 (20.00%)	0 (0.00%)	
Do you check your requested radiology images? ^b^					
	No	6 (8.00%)	0 (0.00%)	0 (0.00%)	0.035^a^
	Yes (before reading the report)	48 (64.00%)	23 (79.30%)	0 (0.00%)	
	Yes (after reading the report)	21 (28.00%)	6 (20.70%)	2 (100.0%)	
Have you ever made an avoidable medical error in patient care that could have been prevented by reading the radiology report more carefully?					
	No, never	49 (65.30%)	18 (60.00%)	1 (50.00%)	0.659
	Yes, once in my entire career	12 (16.00%)	3 (10.00%)	1 (50.00%)	
	Yes, 2-5 times	10 (13.30%)	6 (20.00%)	0 (0.00%)	
	Yes, >5 times	4 (5.30%)	3 (10.00%)	0 (0.00%)	

Specialists were significantly more likely to read the entire radiology report compared to consultants and residents (87% vs 55% vs 40.9%, P = 0.006). However, comparing different professional levels, the residents were significantly more likely to never make an avoidable medical error that could have been prevented by carefully reading the report (45% vs 69.6% vs 77.3%, P = 0.019). Moreover, we noted no statistically significant differences based on how often clinicians read the radiology report (P = 0.742) or check the requested images (P = 0.29) (Table 5).

**Table 5:** Stratification of clinicians’ behavior toward the radiology report by different professional level ^a^Chi-square test with P < 0.05 being statistically significant. ^b^Data for one participant was missing.

Variable	Sub-category	Level			P-value
		Consultant	Specialist	Resident	
How often do you read the radiology report?					
	Every requested examination	31 (77.50%)	17 (73.90%)	36 (81.80%)	0.742
	Occasionally	9 (22.50%)	6 (26.10%)	8 (18.20%)	
Which part of the radiology report you read?					
	Only conclusion	4 (10.00%)	0 (0.00%)	3 (6.80%)	0.006^a^
	The entire report	22 (55.00%)	20 (87.00%)	18 (40.90%)	
	The conclusion and scan through the text	14 (35.00%)	3 (13.00%)	23 (52.30%)	
Do you check your requested radiology images? ^b^					
	No	1 (2.60%)	0 (0.00%)	5 (11.40%)	0.29
	Yes (before reading the report)	28 (71.80%)	16 (69.60%)	27 (61.40%)	
	Yes (after reading the report)	10 (25.60%)	7 (30.40%)	12 (27.30%)	
Have you ever made an avoidable medical error in patient care that could have been prevented by reading the radiology report more carefully?					
	No, never	18 (45.00%)	16 (69.60%)	34 (77.30%)	0.019^a^
	Yes, once in my entire career	6 (15.00%)	5 (21.70%)	5 (11.40%)	
	Yes, 2-5 times	11 (27.50%)	1 (4.30%)	4 (9.10%)	
	Yes, >5 times	5 (12.50%)	1 (4.30%)	1 (2.30%)	

The professional level significantly affected which part of the radiology is being read (P = 0.006). Finally, due to the small number of participants in each specialty, such as intensive care unit (ICU), ear, nose and throat (ENT), neurosurgery, ophthalmology, and plastic surgery (1, 3, 1, 1 and 2, respectively), making inferential statistics was inapplicable. The differences in making avoidable medical errors that could have been prevented by carefully reading the radiology report were then pooled against which part of the radiology report is most often read. However, we noted no statistically significant differences between the different groups (P = 0.749) (Table 6). Furthermore, changes in the behaviors of reading the radiology report or committing medical errors based on specialty are presented in the appendices (Table 7).

**Table 6:** Changes in making avoidable medical errors by different parts of the radiology report

			Have you ever made an avoidable medical error in patient care that could have been prevented by reading the radiology report more carefully?				Total	P-value
			No, never	Yes, once in my entire career	Yes, 2-5 times	Yes, >5 times		
Which part of the radiology report you read?	Only conclusion	N	6	0	1	0	7	0.749
		%	85.7%	0.0%	14.3%	0.0%	100.0%	
	The entire report	N	35	11	9	5	60	
		%	58.3%	18.3%	15.0%	8.3%	100.0%	
	The conclusion and scan through the text	N	27	5	6	2	40	
		%	67.5%	12.5%	15.0%	5.0%	100.0%	
Total		N	68	16	16	7	107	
		%	63.6%	15.0%	15.0%	6.5%	100.0%	

## Discussion

The radiology report is usually the communication method between radiologists and clinicians. Therefore, it is crucial for radiology reports to be sufficient and adequate enough to fulfill their clinical question. In our study, we asked the clinicians about their behavior of reading the requested radiographic reports and reviewing the images. We wanted to determine how often clinicians read the radiology report, which parts of the report they read the most, how frequently they check requested images, and how frequently they make medical errors that could have been avoided by carefully going through the radiology report. Moreover, we wanted to determine whether these behaviors change based on a set of demographic characteristics, including age, gender, professional level, and specialty.

The majority of our population (78.5%) reported reading the report for every requested examination. However, only 56.1% of the entire population reported reading the full report and 37.4% of participants stated that they read the conclusion and skim through the report for further information regarding the findings. Also, 66.4% of the participants checked the requested images before reading the text of the report. The majority of participants (63.6%) had not committed any errors in relation to not reading the report. A similar study was conducted in 2013 on 102 clinicians. The study showed that 69% of clinicians read the entire report while only 33% of clinicians admitted to committing one or more medical errors that could have been prevented by reading the entire report [6]. Furthermore, a similar research study published in 2018, investigated the opinion of the referring physician with regards to the radiology report on 70 healthcare worker with 45.7% of which were clinicians [1]. The study reported that 55.7% of referring physicians read the radiology report in full, which is almost the same as our reported percentage of 56.1%. Moreover, in the previous study, 67.1% of physicians preferred that the radiology report would be structured, and 75.7% preferred if the conclusion section of the report would incorporate a list of the various diagnostic possibilities. Moreover, a research study was conducted at a public and a university hospital to evaluate the expectations of clinicians. The study showed that the majority of clinicians (70.5%) preferred if a recommendation section was provided at the end of the report, which would have helped them understand the report more [5,7]. This would significantly help clinicians easily reach the definitive diagnosis by combining the data of the radiology report with the clinical presentation and examination of each patient [1,8,9]. Based on the aforementioned observations, it is important to form a relevant and convenient conclusion section in the radiology report, given that a representative number of clinicians do not read the report fully, as we found in our research that 6.5% of the physician read only the conclusion.

In this study, we found that female physicians were more likely to read the full radiology report (56.80%) compared to male peers (55.60%). We also noted that physicians within the age group of 40-60 years were significantly more likely to read the entire report and to check the requested images before reading the report text compared to other age groups (20-39 or >60 years). Specialists, in our study, were significantly more likely to read the radiology report in full; however, residents were significantly more likely to never commit any medical errors that could have been easily avoided by carefully going through the radiology report. This can be attributed to the fact that they are more careful in reading reports as they are still under training. Some studies suggest that the role of the training program itself can contribute to decreasing medical errors in general [10-12]. Naveh et al. stated that decreasing medical errors, in general, is reduced to the fact that residents tend to seek more senior help [13].

Lastly, the age of the clinicians had a significant impact on reading the radiology report where the older the clinician is the less likely he/she will read the entire report. This can be put in line with the reported study that was done in 2011. The study reported that attending physicians, who their mean age was 76.5 and 52 for males and females, respectively, spent less time reading the entire report in comparison to residents [14]. This can further support the fact that residents under training try to avoid medical errors as much as possible due to their limited experience compared to seniors. Interestingly, the age of the practitioner was also significant as the age group of 40-60 years of age were more likely to check the requested radiology image prior to reading the report compared to age groups 20-39 and >60 years (P = 0.035). This is yet to be explored for future studies as to the best of our knowledge no other study explored this area. However, we hypothesize that at the early career stages, clinicians often learn from their mistakes and start paying more attention to details until they reach a peak of report reading in the middle stage of their careers. Afterward, it starts declining as experience plays a role in gaining more confidence in reading the reports.

Due to the significant diversity of specialties of included participants, we couldn’t make inferential statistics related to the differences in behaviors toward report reading. We also examined whether the frequency of committing medical errors would have changed according to which part of the radiology report is often read by the referring physician. However, we found no statistically significant differences among the studied groups.

Despite the fact that some diagnoses may be missed due to some technical or physical limiting factors related to the imaging modality, including imaging resolution, contrast, and signal-to-noise ratio, the majority of errors are attributable to under-reading of the radiology report is (42%) [15,16]. In our study, we noted that 43.9% of physicians read the conclusion section only or skim through the report for relevant findings. This may lead to a significant increase in the rate of attributable medical errors. We also noted that 36.5% of our population committed medical errors (more than once) that could have been easily avoided by carefully going through the report.

Therefore, based on our study's results it is recommended to read the entire radiology report prior to initiating the treatment plan. Also, writing the conclusion of the report in very short and brief sentences might motivate clinicians to read the whole report. Furthermore, writing phrases in the summary such as see above will also give physicians reason to go through the entire report. Lastly, eliminating the conclusion or making it not sufficient enough should be studied further in the future.

Even though we performed epidemiological analyses based on age, gender, professional level, and specialty to examine any disparities among them, our study has certain limitations. The most important of which is the relatively small sample size of our study which took place in one hospital setting. These limitations made it difficult to reach a statistical significance level in most variables. In addition, due to the fact of having a big number of specialties in our study with a low number in each specialty, inferential statistics between specialties was not conducted. This also has limited our study in exploring the relationship between each specialty behavior. Furthermore, similar studies have been more likely to explore the structure of the report rather than the behavior itself so we couldn’t compare a lot of our results with the literature. Therefore, our findings should be interpreted with caution and not be considered representative of the general population. Prospective studies with larger sample sizes are still warranted to confirm our findings.

## Conclusions

In conclusion, gender, age, and training level played a big role in affecting the behavior toward how a radiology report is read. Building awareness toward clinicians on the importance of reading the whole radiology report is necessary to avoid medical errors in the future. However, more studies should be carried out to compare the behaviors of clinicians from various specialties toward reading the radiology report.

## Appendices

**Table 7:** Stratification of clinicians’ behavior towards the radiology report by specialty ^a^Data of one participant is missing. OB & Gyn: obstetrics and gynecology; ENT: ear, nose and throat; ICU: intensive care unit

Variable	Sub-category	Specialty																P
		General Surgery	OB & GYN	Orthopedic	ENT	ICU	Pediatrics	Internal Medicine	Anesthesia	Emergency	Neurosurgery	Urology	Cardiothoracic	Plastic Surgery	Ophthalmology	Family Medicine	Others	
How often do you read the radiology report?																		
	Every requested exam	14 (93.30%)	7 (87.50%)	2 (40.00%)	3 (100%)	1 (100%)	21 (80.80%)	17 (85.00%)	2 (66.70%)	7 (70.00%)	0 (0.00%)	1 (50.00%)	0 (0.00%)	2 (100.00%)	1 (100.00%)	2 (66.70%)	4 (80.00%)	NA
	Occasionally	1 (6.70%)	1 (12.50%)	3 (60.00%)	0 (0.00%)	0 (0.00%)	5 (19.20%)	3 (15.00%)	1 (33.30%)	3 (30.00%)	1 (100.00%)	1 (50.00%)	2 (100.00%)	0 (0.00%)	0 (0.00%)	1 (33.30%)	1 (20.00%)	
Which part of the radiology report you read?																		
	Only Conclusion	1 (6.70%)	1 (12.50%)	0 (0.00%)	1 (33.30%)	0 (0.00%)	1 (3.80%)	0 (0.00%)	0 (0.00%)	1 (10.00%)	0 (0.00%)	0 (0.00%)	1 (50.00%)	0 (0.00%)	0 (0.00%)	1 (33.30%)	0 (0.00%)	NA
	The entire report	11 (73.30%)	5 (62.50%)	4 (80.00%)	0 (0.00%)	1 (100%)	14 (53.80%)	8 (40.00%)	2 (66.70%)	5 (50.00%)	1 (100%)	2 (100%)	1 (50.00%)	2 (100.00%)	0 (0.00%)	2 (66.70%)	2 (40.00%)	
	The conclusion and scan through the text	3 (20.00%)	2 (25.00%)	1 (20.00%)	2 (66.70%)	0 (0.00%)	11 (42.30%)	12 (60.00%)	1 (33.30%)	4 (40.00%)	0 (0.00%)	0 (0.00%)	0 (0.00%)	0 (0.00%)	1 (100%)	0 (0.00%)	3 (60.00%)	
Do you check your requested radiology images?^a^																		
	No	1 (6.70%)	1 (12.50%)	0 (0.00%)	0 (0.00%)	0 (0.00%)	2 (7.70%)	1 (5.00%)	1 (33.30%)	0 (0.00%)	0 (0.00%)	0 (0.00%)	0 (0.00%)	0 (0.00%)	0 (0.00%)	0 (0.00%)	0 (0.00%)	NA
	Yes (Before reading the report)	11 (73.30%)	2 (25.00%)	5 (100%)	2 (66.70%)	1 (100%)	17 (65.00%)	13 (65.00%)	2 (66.70%)	8 (80.00%)	1 (100%)	2 (100%)	2 (100%)	0 (0.00%)	1 (100%)	2 (66.70%)	2 (50.00%)	
	Yes (After reading the report)	3 (20.00%)	5 (62.50%)	0 (0.00%)	1 (33.30%)	0 (0.00%)	7 (26.90%)	6 (30.00%)	0 (0.00%)	2 (20.00%)	0 (0.00%)	0 (0.00%)	0 (0.00%)	2 (100%)	0 (0.00%)	1 (33.30%)	2 (50.00%)	
Have you ever made an avoidable medical error in patient care that could have been prevented by reading the radiology report more carefully?																		
	No, never	11 (73.30%)	5 (62.50%)	5 (100%)	3 (100%)	0 (0.00%)	16 (61.50%)	11 (55.00%)	2 (66.70%)	3 (30.00%)	1 (100%)	1 (50.00%)	2 (100%)	1 (50.00%)	1 (100%)	3 (100%)	3 (60.00%)	NA
	Yes, once in my entire career	3 (20.00%)	0 (0.00%)	0 (0.00%)	0 (0.00%)	1 (100%)	2 (7.70%)	6 (30.00%)	1 (33.30%)	1 (10.00%)	0 (0.00%)	1 (50.00%)	0 (0.00%)	1 (50.00%)	0 (0.00%)	0 (0.00%)	0 (0.00%)	
	Yes, 2-5 times	1 (6.70%)	1 (12.50%)	0 (0.00%)	0 (0.00%)	0 (0.00%)	6 (23.10%)	2 (10.00%)	0 (0.00%)	4 (40.00%)	0 (0.00%)	0 (0.00%)	0 (0.00%)	0 (0.00%)	0 (0.00%)	0 (0.00%)	2 (40.00%)	
	Yes, >5 times	0 (0.00%)	2 (25.00%)	0 (0.00%)	0 (0.00%)	0 (0.00%)	2 (7.70%)	1 (5.00%)	0 (0.00%)	2 (20.00%)	0 (0.00%)	0 (0.00%)	0 (0.00%)	0 (0.00%)	0 (0.00%)	0 (0.00%)	0 (0.00%)	
